# Double-Edged Sword Effect of Diet and Nutrition on Carcinogenic Molecular Pathways in Breast Cancer

**DOI:** 10.3390/ijms252011078

**Published:** 2024-10-15

**Authors:** Anca-Narcisa Neagu, Claudiu-Laurentiu Josan, Taniya M. Jayaweera, Krishan Weraduwage, Niyogushima Nuru, Costel C. Darie

**Affiliations:** 1 Laboratory of Animal Histology, Faculty of Biology, “Alexandru Ioan Cuza” University of Iași, Carol I bvd. 20A, 700505 Iasi, Romania; claudiujosan22@gmail.com; 2 Biochemistry & Proteomics Laboratories, Department of Chemistry and Biochemistry, Clarkson University, Potsdam, NY 13699-5810, USA; jayawetm@clarkson.edu (T.M.J.); weraduk@clarkson.edu (K.W.); nurun@clarkson.edu (N.N.)

**Keywords:** breast cancer (BC), breast cancer risk (BCR), diet, nutrition, carcinogenic molecular pathways, bioactive compounds, food contaminants

## Abstract

Environmental exposure to a mixture of chemical xenobiotics acts as a double-edged sword, promoting or suppressing tumorigenesis and the development of breast cancer (BC). Before anything else, we are what we eat. In this review, we highlight both “the good” and “the bad” sides of the daily human diet and dietary patterns that could influence BC risk (BCR) and incidence. Thus, regularly eating new, diversified, colorful, clean, nutrient-rich, energy-boosting, and raw food, increases apoptosis and autophagy, antioxidation, cell cycle arrest, anti-inflammation, and the immune response against BC cells. Moreover, a healthy diet could lead to a reduction in or the inhibition of genomic instability, BC cell stemness, growth, proliferation, invasion, migration, and distant metastasis. We also emphasize that, in addition to beneficial compounds, our food is more and more contaminated by chemicals with harmful effects, which interact with each other and with endogenous proteins and lipids, resulting in synergistic or antagonistic effects. Thus, a healthy and diverse diet, combined with appropriate nutritional behaviors, can exert anti-carcinogenic effects and improve treatment efficacy, BC patient outcomes, and the overall quality of life of BC patients.

## 1. Introduction

BC is the most diagnosed cancer worldwide, with over 2.3 million new cases and 685,000 deaths in 2020; BC diagnosis is predicted to increase to over 3 million new cases and one million deaths by 2040 [1]. Evidence suggests that environmental exposure to certain chemicals and lifestyle account for 70% to 90% of the risk factors for chronic diseases, whereas only 10% to 30% can be explained by a specific genomic landscape [2]. Both dietary compounds and daily nutrition-related habits may play key roles in preventing diseases [3] or, on the contrary, can exert carcinogenic effects, even at low levels of exposure [4]. The expression a “double-edged sword” refers to something that has both good and bad consequences. For example, food contaminants are usually chemical substances, as well as microbial or physical compounds present in edibles, which may be harmful to human health, with different levels of severity [5]. Moreover, food contaminants are generally environmental pollutants, which are not present naturally in raw food, with contamination occurring during the production, processing, distribution, storage, packaging, transportation, or preparation of food. On the contrary, a plethora of bioactive compounds are present in “functional food”, dietary supplements, and plant or animal nutraceuticals, which are known to exert beneficial effects on health [6].

Phytochemistry, as well as various omics fields, such as nutrigenomics, nutriproteomics, metabolomics, interactomics, and exposomics [7], offer a wide range of opportunities for the development of personalized diets for women at risk of developing BC [3]. It was shown that certain dietary compounds, mainly phytonutrients, can modulate gene expression, acting on the main cancer hallmarks: cell growth and proliferation, genome instability and mutagenesis, angiogenesis, metabolism reprogramming, anti-apoptosis, tumor-promoting inflammation, therapy resistance, invasion, and metastasis [8]. Plant-based diets may contribute to the inhibition of BC cell proliferation, differentiation, invasion, metastasis, angiogenesis, and anti-apoptotic mechanisms, by targeting numerous molecular pathways and promoting beneficial effects in terms of the prevention and treatment of BC [9]. Moreover, plant-based dietary bioactive compounds can modulate hormone-induced reactions, genetic and epigenetic regulation, and promote the activation of oxidative stress (OS) and endoplasmic reticulum stress, triggering redox reactions, as well as the aggressive behavior of tumor cells caused by the production of reactive oxygen species (ROS) [10]. Many types of food rich in bioactive compounds, such as polyphenolic compounds, carotenoids, terpenoids, and sulfur-containing compounds, have been recommended as chemopreventive and chemotherapeutic agents, including asparagus, broccoli, Brussels sprouts, kale, and other cruciferous vegetables, carrots, grapefruit, soy, spinach, and tomatoes, mainly for ER-negative BC [11]. On the other hand, a plethora of risk factors, such as alcohol consumption [12], added sugar in foods [13], and LDL cholesterol [14], stimulate inflammation, BC cell proliferation, invasion, migration, and metastasis. Moreover, nowadays, more than ever, more food is processed and contaminated with man-made chemicals that act as endocrine disruptors, accumulate in high-fat tissues, and stimulate BC initiation and progression.

There are dietary patterns, such as the typical Western diet (WD), characterized by a high intake of pre-packaged foods, refined grains, red and processed/ultra-processed meat, candy, cookies, high-sugar drinks, fried foods, high-saturated fat and high-fructose products, dairy products, alcohol, salt, sugar, sweeteners, and other additives [15]. Thus, the WD has been linked to the development of excess weight/obesity [16], the alteration of human gastrointestinal microbiota [17,18], and carcinogenesis [19]. In postmenopausal obese women, the risk of developing BC is increased [20], while gut microbiota dysbiosis plays a role in the development of BC through estrogen-dependent pathways and microbial-derived metabolites [21]. Conversely, the Mediterranean diet (MD) involves a plant-based dietary pattern, based on a high intake of olive oil and plant-based foods, such as vegetables, non-refined cereals, legumes, and nuts, and a moderate or low level of consumption of dairy and meat-based products, alcohol, and sweets [22]. Consequently, the MD could reduce BCR and enhance survival through its anti-inflammatory effects, antioxidant characteristics, and hormone–receptor interactions [22]. Kalam et al. (2023) showed that diet and diet-related behaviors can modulate carcinogenesis, cancer progression, treatment efficacy, and recurrence [23]. Post-diagnosis plant-based diets can also benefit prognosis in cancer patients [24], due to the bioactive compounds that occur in small quantities in foods and that may have beneficial effects on health [25]. In this review, we highlight “the good” and “the bad” sides of the daily human diet and dietary patterns that could influence BC risk (BCR) and incidence. We conclude that a balanced and personalized dietary structure, combined with appropriate nutritional behaviors, can improve treatment efficacy and BC patient outcomes.

## 2. The Good

Circadian nutritional behaviors have been associated with BC [26] and, when disrupted, become factors for BCR [27]. Moreover, eating more frequently, reducing evening energy intake, and fasting for longer intervals, can downregulate the biomarkers of inflammation, as well as BCR [28]. Breakfast is the first meal of the day, often considered to be “the most important meal of the day” [29]; evidence shows that a good quality breakfast and breakfast-based nutrients are beneficial for human health, in association with a lower body mass index (BMI), a higher level of satisfaction in life, and a higher intellectual performance [30,31,32,33,34,35,36,37]. However, Elahy et al. (2023) concluded that there are no associations between the frequency of breakfast meals or after-dinner snack habits and BCR in postmenopausal women [38]. It is known that cyclical fasting or fasting-mimicking diets enhance antitumor chemotherapy effects in TNBC models [39], as well as the activity of endocrine therapeutics in mouse models of hormone receptor-positive BC [40]. A systematic review conducted by Anemoulis et al. (2023) concluded that intermittent fasting reduces chemotherapy-induced DNA damage in BC patients [41]. These nutritional patterns combined with specific therapeutic drugs reduce circulating insulin growth factor 1 (IGF1), insulin and leptin, and inhibit AKT/mTOR signaling through the upregulation of early growth response protein 1 (EGR1) and the phosphatase and tensin homolog deleted on chromosome 10 (PTEN), thus promoting long-lasting tumor regression and reverting the acquired resistance to chemotherapy [40]. Thus, intermittent fasting, based on different patterns of time-restricted feeding behaviors, attenuates obesity-induced TNBC progression in cell and animal models, involving multiple pathways, such as those involved in EMT reduction, cell cycle disruption, reductions in systemic glucose and cholesterol levels, and the downregulation of inflammatory factors in the TME [42]. A multicase control study in Spain showed that having breakfast at a later time of day was associated with a non-significant increase in BCR [26].

Among the diverse dietary patterns, the Mediterranean diet, rich in antioxidants and anti-inflammatory compounds; the plant-based dietary pattern, rich in fibers, phytochemicals, lignans, carotenoids, vitamins C and E, folate, and phenolic acid; the prudent dietary pattern, rich in spices, plant-based oils, low-fat dairy, and seafood; the healthy dietary pattern, rich in fruits, vegetables, legumes, seeds, and nuts; the ketogenic dietary pattern, known as a high-fat and low-carb diet; the paleolithic dietary pattern, rich in vegetables, fruits, lean meats, fish, nuts, and seeds; and the dietary approaches used to stop hypertension, were all correlated with a lower risk of developing BC [43]. Diverse breakfast patterns have been geographically identified and are based on cereal or sweetened breads and milk, eggs, sweetened beverages, sandwiches, and fruit consumption [30]. In Romania, traditional food products are much more popular than foods purchased from chain stores [44], because local consumers are attracted by traditional and authentic gastronomy [45]. Moreover, the main basic food products consumed are meat and meat products traditionally based on pork/chicken, such as pork crackling, pork fat, grilled pork neck, pork loin, smoked pork knuckle, grilled meat rolls, cabbage rolls with ground pork and rice, chicken liver, deep-fried chicken, pastrami, and sausages, milk and/or milk products (sour cream, cheese), vegetables (fries, beans, mushrooms, smashed potatoes, cabbage, pickles, garlic), and fruits, bread/bakery and pastry products (mamaliga/polenta, noodles, ice cream, fried cheese doughnuts), and fish (mainly fried crap) or fish products [44]. Fast food consumption is more popular among children and adolescents [44]. There are studies that have suggested that certain foods are consumed in excess (products high saturated fat, cholesterol, salt, sugar, refined grains, and alcohol), while there is a deficiency in regard to the intake of nutritional factors (essential amino acids, polyunsaturated fatty acids, vitamins C, A, B, D, folic acid, calcium, and iron) [46].

These meals contain complex mixtures of natural and added chemical compounds that can exert double-edged sword effects on carcinogenic molecular pathways in breast tissue. For example, milk is a complex biofluid, rich in primary nutrients and is considered a complete and basic food that is consumed by billions of people worldwide [47]. Thus, pure cow’s milk and its derivative dairy products, such as cream, butter, cheese, and yogurt are a major sources of nutrition in the human diet [48]. Wajszczyk et al. (2021) showed that individual dairy products have a statistically significant, but bi-directional relationship with BCR, which was different for premenopausal and postmenopausal Polish women [49]. A cohort study conducted by these authors concluded that an increase in consumption of one serving of dairy products/week may significantly decrease the BCR, by 2%, for premenopausal women only, while cottage cheese consumption significantly reduced the BCR by 20%, following an increase of one serving/week, for postmenopausal women only [49]. A 5% decrease in BCR has been observed as a result of an increase in dairy consumption of one glass of milk/week in both strata of women during the menopause [49]. Arafat et al. (2023), after performing a systematic literature review, also concluded that dairy consumption was inversely associated with BCR, even when the effects of different types of dairy products and the dose–response relationship on BCR remain unknown [50]. More recently, Riseberg et al. (2024) showed that the overall amount of dairy consumption was not associated with BCR, but the results can vary according to the tumor subtype, and heterogeneity was observed in terms of the type of dairy food and the patient’s period of life [51]. Deschasaux-Tanguy et al. (2022) found no association between the consumption of dairy products or dairy desserts high in sugar and BCR [52].

Almost 70% of the fat in milk is saturated, 25% is monounsaturated, and 2.3% is polyunsaturated, with a variable omega-6/omega-3 ratio [53]. Moreover, α-linolenic acid (ALA), an omega-3 fatty acid, induces apoptosis and inhibits invasion, metastasis and angiogenesis, and arrests the cell cycle in human BC cells by inhibiting fatty acid synthase (FASN), which is usually overexpressed in various cancers [54,55]. Conjugated linoleic acid (CLA) is known as a group of isomers of linoleic acid (LA), of which cis-9 trans-11 (c9,t11-CLA) is the one that has the highest percentage, being produced through biohydrogenation by lactic acid bacteria in the rumen or by endogenous synthesis in the mammary gland [56]. Consequently, CLA is present in the milk, dairy, and meat products of ruminants, with dairy products being the principal source of CLA in the human diet [57]. Moreover, 3 g/day of CLA is the recommended intake, with beneficial effects on human health [58], in principal due to the antitumor effects of CLA [56]. Zeng et al. (2019) demonstrated that dietary intake of c9,t11-CLA enriched from butter reduces BC progression in vivo via the downregulation of the progesterone receptor (PR) and Ki-67 expression [57]. Milk proteins, which include 17–20% whey proteins (α-lactalbumin, β-lactoglobulin, glycoproteins, lactoferrin, immunoglobulins, peptide hormones, enzymes like lactoperoxidase, and serum albumin) and 78–80% casein (αs-1, αs-2, β-, and k-casein), as well as milk peptides, exert many biological proprieties, such as antibacterial, antiviral, antifungal, and antioxidant activities [59]. Using cow’s milk bottom-up proteomics, based on combinatorial SDS-PAGE profiling and trypsin digestion, followed by nanoHPLC-electrospray ionization tandem mass spectrometry (nLC-ESI-MS/MS), Vincent et al. (2016) identified 186 different major and minor proteins, including β2-microglobulin (β2-M), osteopontin (OPN), lipoprotein lipase (LPL), sulfhydryl oxidase (SOX), xanthine dehydrogenase/oxidase (XOR), β-1,4-galactosyltransferase 1 (Gal-T1), lactadherin/milk fat globule-EGF factor 8 (MFG-E8), lactotransferrin, mucins 1 and 15, α-1-acid glycoprotein, α-1B-glycoprotein, α-2-HS-glycoprotein, pancreatic secretory granule membrane major glycoprotein GP2, platelet glycoprotein 4, Zn-α-2-glycoprotein, milk glycosylation-dependent cell adhesion molecule 1/lactophorin (GlyCAM1), dystroglycan (DG), and peptidoglycan recognition protein 1 [48]. Some reports suggest that OPN may exhibit antitumorigenic characteristics in certain circumstances [60]. Orally administrated lactoferrin (LF), a natural proapoptotic iron-binding multifunctional glycoprotein from bovine milk, belonging to the transferrin family, can exert strong anticancer activities [61]. Pereira et al. (2016) showed that bovine LF preferentially induces apoptosis in the highly metastatic BC cell lines, Hs 578T and MDA-MB-231, but not in the less metastatic T47D or in the non-tumorigenic MCF10A cell lines [62]. The authors demonstrated that LF decreases the extracellular acidification rate and causes intracellular acidification in metastatic BC cells through the inhibition of plasmalemmal V-H^+^-ATPase, which transports protons across cellular membranes [62].

Milk is also an abundant source of extracellular vesicles (EVs); bovine milk-derived extracellular vesicles (MEVs) are able to sensitize TNBC cells into doxorubicin by targeting metabolism and STAT signaling pathways, thus reducing the abundance of many tumorigenic proteins associated with a worse prognosis and low overall survival in TNBC [63]. Samuel et al. (2021) showed that orally administrated MEVs survive the degrading conditions in the mouse gut and can be detected in various organs, while MEVs implanted in BC cells reduced the primary tumor burden, but accelerated metastasis in BC mouse models through the induction of EMT, providing context-based and opposing roles of MEVs as metastasis promoters and suppressors [64]. Ramezani et al. (2023) showed that bovine milk lactoferrin-loaded exosomes induce selective toxicity against BC cells compared to normal cells and that incorporating lactoferrin into exosomes could have an antitumorigenic role through inducing the overexpression of the proapoptotic BH3 interacting domain death agonist (BID) protein and diminishing the expression of the anti-apoptotic protein Bcl2 in the human MDA-MB-231 BC cell line, following exoLF treatment [65]. Shariatikia et al. (2017) showed that mare, donkey, cow, and camel milk, and their casein and whey proteins, have potent cytotoxic effects against the MCF7 BC cell line in a dose-dependent manner, while sheep and goat milk and their proteins did not exert any cytotoxic activity [59].

A comprehensive meta-analysis of prospective cohort studies concluded that fermented milk-derived products, including yogurt and sour milk, were associated with lower cancer mortalities [66]. However, another meta-analysis conducted by Chen et al. (2019) concluded that low-fat/skimmed milk, whole milk, and yogurt intake had no effect on BCR [67]. To sustain this idea, a pooled analysis of 21 cohort studies conducted by Wu et al. (2021) showed that dietary calcium consumption was not clearly associated with BCR, but higher yogurt and cottage/ricotta cheese intake were found to be inversely correlated with the risk of ER-negative BC [68]. Cheese is one of the fermented dairy products rich in proteins, minerals, organic acids, bioactive peptides, oligosaccharides, and vitamins, and also contains diverse non-pathogenic microorganisms that act as probiotics [69]. Kamal and Talib (2023) showed that a combination of the ketogenic diet, which is high in fat, low in carbohydrates, and sufficient in terms of proteins, and probiotics inhibits BC in mice through the downregulation of IGF-1 and immune system modulation [70]. Ryser et al. (2022) showed that a gram-negative bacterium, *Morganella morganii*, isolated from the outer layers of raclette-type cheese, influences the formation of biogenic amines in cheese, such as cadaverine and putrescine, which is undesirable, since their consumption can cause intoxication [71]. Interestingly, treatment of BC cell lines with cadaverine according to its serum reference range reverted ETM, decreased cell motility and invasion, and inhibited cell stemness by reducing mitochondrial oxidation [72]. Moreover, Ritota et al. (2022) emphasized that bioactive compounds, such as picrocrocin/safranal and crocin, from cow and ewe cheeses made with saffron, usually used to add a natural yellow to orange color to cheeses, exert antiproliferative effects in regard to MDA-MB-231 BC cells [73].

Nondairy/plant-based substitutes for cow’s milk and its derivatives, manufactured from soy, rice, coconut, and almonds, have gained in popularity in recent years, with adoption of vegetarian and vegan diets [74]. In the USA, the proportion of individuals reporting that they consumed soy milk was 1.54% in 2017–2020 [75]. Moreover, non-Hispanic Asian and Black ethnicities have significantly increased the consumption of soy milk [75]. Soy milk and its derivatives contain many natural isoflavones that are similar to estrogen hormones, known as phytoestrogens [76], so the beneficial effects of soy food intake remain controversial [77]. There is a hypothesis that suggests that soy isoflavones, genistein and daidzein, may stimulate the proliferation of ER+ BC cells, even at low concentrations [77]. However, genistein, a natural isoflavonoid, is also considered to be a potent anti-BC agent, usually present in high quantities in soybeans, inducing antiproliferative effects/arrest of the cell cycle and apoptosis, and preventing tumor angiogenesis [78]. In different brands of commercially available soy milk, the mean genistein content is 17.58 ± 8.38 µg/mL [76]. At high concentrations, genistein kills MCF7 BC cells [79] or delays TNBC tumor growth [80], inhibiting proliferation/differentiation and inducing apoptosis [81,82]. Genistein also inhibits angiogenesis [83], induces tamoxifen resistance and growth in ER+/HER2+ BC cells, and inhibits the growth of ER-/HER2+ BC cells [84]. At the molecular level, genistein suppresses the IGF-1R/p-AKT signalling pathway; decreases the Bcl-2/Bax ratio [81]; downregulates the NF-κB/Bcl-xL/TAp63 signaling pathway; induces the modification of key epigenetic cancer-associated genes, their enzymatic activities, genomic DNA, and histone methylation [80]; upregulates PI3K and MAPK signalling; and downregulates p27^Kip1^ levels in ER+/HER2+ BC cells [84]. In addition, daidzein, an isoflavone from fruits, nuts, soy beans, and soy-based products [85], inhibits BC cell proliferation, induces apoptosis [85,86], and inhibits TNF-α-induced migration and invasion [87]. The antineoplastic effects of daidzein are mediated by cell cycle arrest, the inhibition of cyclin D and CDK2/4, and increases in p21^Cip1^, p57^Kip2^ expression, and caspase-9 activity [85]. Moreover, daidzein generates ROS, disrupts mitochondrial function, decreases cyclin-D expression [86], inhibits TNF-α, suppresses Hedgehog/GLI1 signalling [87], upregulates Bax, downregulates Bcl-2, induces apoptosis, lowers the ERα/β ratio, and increases ROS production [88].

Meat has been a part of the human diet since the early stages of our existence, with archeological findings suggesting that food was cooked for the first time around 300,000–400,000 years ago (some 7000–14,000 generations ago) [89]. Even if meat consumption is correlated with an increased BCR, Lo et al. (2019) showed that poultry consumption may be associated with reduced risk, so substituting poultry for red meat could decrease BCR [90].

Grains are one of the most important foods consumed worldwide that contain bioactive compounds, mainly found in whole grain cereals, such as wheat, rye, oats, and barley [91,92]. A meta-analysis conducted by Xiao et al. (2018) concluded that high intake of whole grains might be inversely associated with BCR in case-control studies, but not in cohort studies [91]. However, carbohydrate-based foods with high glycemic index may influence BCR via the insulin growth factor axis [93]. The contamination of cereals and cereals-based products with mycotoxins, such as aflatoxins, has been associated with mutagenesis and carcinogenesis [94,95]. Aljazzar et al. (2022) showed that aflatoxin B1 (AFB1) causes significant toxicity in regard to MCF7 BC cells through oxidative stress (OS), as well as transcriptomic alterations in regard to drug-metabolizing enzymes, transporters, and antioxidant enzymes as a result of in xenobiotics [95]. Moreover, AFB1 upregulates pro-inflammatory markers, such as tumor necrosis factor alpha (TNFα), cytokine, and cyclooxygenase-2, correlated with the reduction in the mRNA expression of immunity-related genes, including interleukins 8 and 10 [95]. In BC, TNFα has a pro-tumorigenic role and correlates with increased proliferation, a higher malignancy grade, metastasis, and poor prognosis [96,97], due to the ability of TNFα to upregulate TAZ, a transcriptional co-activator that promotes BCSC self-renewal in human BC cell lines [98]. Dietary baker’s yeast, commonly used in baking bread and other bakery products, can enhance the apoptotic ability of paclitaxel against the human MCF7 BC cell line and the metastatic murine 4T1 cell line [99]. A systematic review conducted by Grudzinska et al. (2023) emphasized that dietary sprouts may play a role in the chemoprevention of BC [100]. Thus, germinated wheat flour reduces the growth of MCF7 and MDA-MB-231 human BC cell lines, upregulating apoptosis [101].

Eggs are also frequently consumed worldwide as a nutrient-rich food, due to their protein and peptide content [102]. Due to advancements in MS-based proteomics, 167 proteins were identified in the egg white proteome, which varies based upon the storage of the eggs at different temperatures over different time spans, early embryonic development, egg varieties, and stress conditions [103]. Ovalbumin and ovotransferrin have been detected as major egg proteins responsible for multiple bioactivities [103,104]. Thus, the native ovotransferrin (OTRF/OTF)/conalbumin, an iron-binding glycoprotein from the transferrin protein family, present both in avian plasma and egg white [105], is known to have anticancer activities, with negative effects on the proliferation of MCF7 BC cells by inducing apoptosis [104,106].

Nutritional supplements are also consumed at breakfast time [30]. For example, bee pollen is an excellent dietary supplement in regard to human nutrition [107]. Pollen, also known as bee bread (BB), contains various bioactive polyphenolic compounds, such as isoflavonoids (genistein, daidzein, glycitein, biochanin A, formononetin, puerarin, coumestrol, and equol) and flavonoids, like quercetin, kaempferol, apigenin, luteolin, myricetin, hesperetin, rutin, naringenin, catechin, epicatechin, epigallocatechin, and proanthocyanidins [108]. Genistein is the major isoflavone identified in bee pollen [109], interfering with several biological processes, pathways, and genes/proteins, including PTEN, PI3K, PIP3, AKT, mTOR, Bcl-2 Bax, caspase-3, cyclin B, VEGF, HIF1α, p21, and p16, EGFR/AKT/NF-κB, DNA methylation, the ER pathway, and MMP genes [78]. At a concentration of 370 µM, genistein revealed a cytotoxic effect on MCF7, T47D, and MDA-MB-231 BC cell lines, while at a concentration of 0.37 µM, no significant effect on BC cell viability was observed [110]. Synergically, doxorubicin (DOX), cisplatin, and BB, blocked the migration of MDA-MB-231 cells and suppressed the proapoptotic BID gene, overexpressing the anti-apoptotic Bcl-2 gene, reducing the toxicity of chemotherapeutic drugs on this TNBC cell line [111]. Another study found that the synergistic effect of BB with DOX led to the suppression of the proliferation of breast tumors in 4T1 tumor-bearing BALB/C mice and inhibited the oxidative damage of DOX, increasing the expression of apoptotic genes and proteins, the p53 level, as well as serum interferon-γ (IFN-γ), and reducing the estrogen level, Ki-67 and Bcl-2 proliferation biomarkers, nitric oxide, and pro-inflammatory cytokines [108].

Leafy vegetables can be effective in terms of “green chemoprevention” and treating BC [112]. Moreover, broccoli and broccoli sprouts contain many active biochemicals, such as sulforaphane (SFN), a natural organosulfur compound known to counteract the tumorigenic effects of chemical xenobiotics in food and the environment [112]. Kaboli et al. (2020) concluded that SFN reduces NF-κB activity, downregulates apoptosis inhibitors, decreases the activity of histone deacetylases leading to cell cycle arrest, as well as increases the sensitivity of BC cells to chemotherapy [113]. Moreover, SFN is involved in the modulation of gene expression and nuclear factor-erythroid factor 2-related factor 2 (NRF2) antioxidant signaling [113]. Wu et al. (2019) indicated that NRF2 signaling has a double-edged sword effect in regard to cell survival, because the NRF2/KEAP1 pathway exerts anticancer activities, but also activates pro-survival genes and promotes cancer cell proliferation [114]. Moreover, Surh (2021) emphasized that SFN blocks the T cell-mediated immune response necessary for tumor immune surveillance [115]. Recently, Zhang et al. (2022) showed that SFN derived from broccoli, kale, cabbage, cauliflower, garden cress, and mustard [112,113], suppresses the metastasis of TNBC cells by targeting the RAF/MEK/ERK pathway to inhibit the formation of actin stress fibers and TGF-β1-induced BC cell migration, invasion, and metastasis [116].

Evidence suggests that consuming a high amount of onions (*Allium cepa*) and garlic (*Allium sativum*) is protective against BC [117]. Allicin, the major active biocompound present in freshly crushed garlic [118], is also a bioactive organo-sulfur compound [119], able to induce cell cycle arrest and apoptosis in MCF7 and MDA-MB-231 BC cell lines through tumor-suppressor p53 signaling pathway activation [120]. Moreover, Shi et al. (2024) showed that a combinatorial treatment of allicin with doxorubicin (DOX) resulted in better effects in regard to inhibiting proliferation and increasing the apoptosis of MCF7 and MDA-MB-231 DOX-resistant BC cells, than the treatment with DOX or allicin alone [118]. Thus, allicin inactivates the NRF2/HO-1 signaling pathway and improves the DOX sensitivity of BC cells [118]. Onion contains allicin, quercetin, fisetin, and other organo-sulfur compounds, such as diallyl disulfide and diallyl trisulfide [121]. Onion, one of the most popular vegetables in the world, is a major source of quercetin [122,123], a flavonoid abundantly found in plants, vegetables, and fruits, mainly in cruciferous vegetables, grapes, apples, tomatoes, and blueberries [124]. Quercetin inhibits the proliferation, migration, and invasion of 4T1 BC cells, suppressing the IL-6/JAK2/STAT3 signaling pathway and promoting the cytotoxicity of tumor immune cells in the TME [125]. Moreover, quercetin induces apoptosis and suppresses cell proliferation in MCF7 and MDA-MB-231 BC cells, changing endonuclease-G (Endo-G) and the expression of caspases 3/8/9 [124].

It was also shown that lycopene has anticancer activities and that it could be considered as a potentially effective compound in BC prevention and treatment [126,127]. The regulation of oxidative and inflammatory processes, angiogenesis, the induction of apoptosis, the inhibition of cell proliferation and metastasis formation, as well as the modulation of gap junctional intercellular communication, growth factors, and signal transduction pathways, have been the most cited mechanisms in terms of lycopene action [127,128]. Lycopene is a red-colored carotenoid pigment found in tomatoes and tomatoes-based products, red fruits, red carrots, watermelons, red grapefruits, papayas, and apricots, which is known to enhance protection against cancer [127]. Takeshima et al. (2014) showed that lycopene induces ERK1/2 activation, cyclin D1 suppression, and p21 upregulation in MCF7 (ER/PR positive), SK-BR-3 (HER2+), and MDA-MB-468 (TNBC) cell lines [129]. Lycopene inhibits AKT phosphorylation and mTOR in TNBC cells, leading to the upregulation of proapoptotic Bax [129]. Unfortunately, chlorpyrifos (CPF) is an organophosphate insecticide extensively used in the production of tomatoes [130]. Ventura et al. (2019) demonstrated that the concentration of this xenobiotic in the environment alters mammary histology and the hormonal balance in chronically exposed rats, acting as a BCR factor [131]. These authors emphasized that CPF alters HDAC1 mRNA expression, which promotes mammary tumor development [131]. Moreover, CPF acts as an endocrine disruptor that promotes migration, invasion, and the stemness phenotype in 3D cultures of MCF7 and MDA-MB-231 BC cell lines and induces the activation of many BC-related pathways, such as EMT [132].

Resveratrol is a natural dietary polyphenol that affects the expression of several cytokines, caspases, MMPs, adhesion molecules, and growth factors. It modulates the activity of several signalling pathways, such as PI3K/AKT, NF-κB, and Notch, which play crucial roles in carcinogenesis [133,134]. Moreover, resveratrol induces Bax-dependent, but p53-independent, apoptosis in MDA-MB-231 BC cells [135]. Resveratrol exists in 70 types of plants and can inhibit the migration and metastasis of MDA-MB-231 human BC cells by reversing transforming growth factor (TGF)-β1-induced EMT [136]. In vitro, resveratrol can decrease the expression levels of MMP2 and MMP9, fibronectin, α-SMA, P-PI3K, SMAD2, SMAD3, P-SMAD2, P-SMAD3, vimentin, Snail1, and Slug, increasing the expression levels of E-cadherin [136]. In vivo, resveratrol inhibits lung metastasis in mice bearing MDA-MB-231 human BC xenografts [136].

Curcumin is a polyphenol derived from turmeric (*Curcuma longa*) that inhibits breast cancer stem cell (BCSC) properties and cell proliferation and promotes apoptosis in the MCF7 BC cell line [137,138]. This polyphenol inhibits the proliferation of BCSCs through the modulation of several signaling pathways, such as NF-kB signaling, which is known as an important curcumin-regulated pathway [139]. NF-kB signaling is involved in the maintenance of a variety of stem cells [140], including BCSCs, which overexpress components of the NF-kB signaling pathway and have high NF-kB activity levels [141]. Moreover, sonic hedgehog (Shh) and WNT/β-catenin signalling pathways are also crucial in maintaining the stemness of BCSCs, with curcumin decreasing the activity of BCSCs by inhibiting tumor sphere formation and decreasing BCSC biomarkers, such as CD44, ALDHA1, Nanog, OCT4, and SOX2, thus downregulating both the Shh and WNT/β-catenin signalling pathway activities, which results in BCSC inhibition [142]. In addition, nuclear factor erythroid 2-related factor 2 (NRF2) is known to regulate oxidative stress, being involved in the development of cancer stem cells and metastasis [143]. Many authors have demonstrated or reviewed how curcumin activates the NRF2 signaling pathway, inducing cellular protection against oxidative injury [144,145]. Moreover, curcumin has very little toxicity in terms of normal stem cells, but has numerous cytotoxic effects on CSCs, due to the suppression of IL-6, IL-8, and IL-1, which stimulate CSCs [146]. Overall, curcumin could function as a cytotoxic and anti-metastasis agent for BC [142]. These chemopreventive, anticancer, and cytotoxic proprieties are also modulated through the downregulation of oncogenic RAF-1, the suppression of telomerase, and the upregulation of TNF-α and IL-8 genes [138]. In MDA-MB-231 and Hs578T TNBC cells, curcumin inhibits motility and migration, downregulating the expression of the proteins involved in EMT, such as the mTOR and PI3K/AKT signalling pathways [147]. Curcumin also downregulates the mRNA expression of vimentin, fibronectin, and β-catenin, and upregulates E-cadherin mRNA expression levels [148].

**Figure 1:**
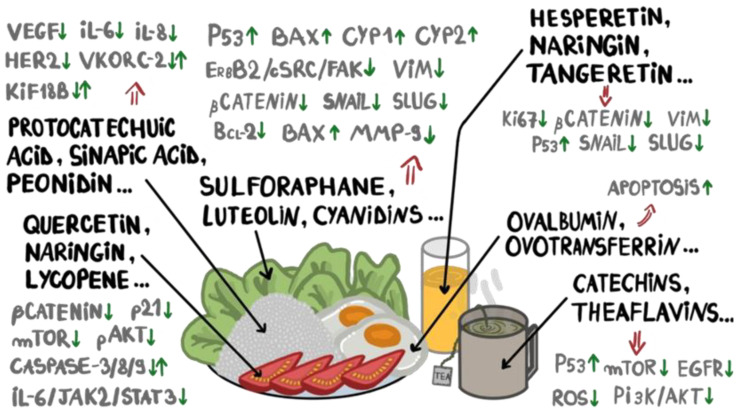
Biomarkers and biological pathways involved in breast cancer initiation and progression, targeted by bioactive compounds present in a daily, diverse diet.

Black and green tea can have chemopreventive effects on BC development, with some authors suggesting that women with a family history of BC should drink about five cups of tea per day in order to decrease the BCR [149]. Flavan-3-ols are a subclass of flavonoids, consisting of polyphenolic phytochemicals found in a wide variety of food sources, especially in fruits and teas [150,151,152,153]. Catechins and theaflavins are both flavan-3-ols with significant bioactive properties, including anticancer, anti-mutagenic, antioxidative, and anti-inflammatory effects [154]. The beneficial effects, at the molecular level, of bioactive compounds that exert anti-BC potential are listed in Figure 1 and Table 1.

## 3. The Bad

The pro-inflammatory diet, based on a high intake of red, processed meat and alcohol [257], was associated with an increased BCR [258], while a long-term anti-inflammatory diet can improve the survival of BC patients [259]. The Western dietary pattern, rich in hydrogenated fat, soft drinks, animal fat, fast food, refined cereals, sweets, and processed meat, and the unhealthy dietary pattern, rich in sugars, processed juices, soft drinks, potato chips and mayonnaise, desserts, solid oils, red and processed meat, and high salt intake, are also associated with increased BCR [43]. Eating breakfast at a later time was associated with increased BCR among premenopausal women [26] and skipping breakfast was associated with an elevated risk in terms of all-cause and cancer-related mortality [260] and seems to be a bad idea for patients with cancer [261]. Moreover, poor diets that include refined sugar, saturated and trans fats, as well as a low level of natural antioxidants and fiber intake, were linked to increased BCR through the modulation of inflammation-related pathways and biomarkers [262] that play a key role in BC initiation and progression [263]. To exemplify this, nuclear factor-kappa B (NF-kB) is a pro-inflammatory nuclear transcription factor and the activation of the NF-kB signaling pathway is common in BC [264]. Alcohol exposure activates the NF-kB pathway and enhances the transcription of NF-kB-targeted genes [12]. Moreover, Starek-Swiechowich et al. (2023) showed that alcohol, even at low concentrations, as well as its major metabolite, acetaldehyde, causes TNBC cell proliferation, migration, and invasion via the activation of p38 MAPK and JNK phosphorylation [12]. Kansestani et al. (2019) showed that high glucose intake increased MCF7 BC cell proliferation, viability, VEGF secretion, and Bcl-2 expression, decreasing apoptosis, and stimulating angiogenesis, due to the activation of the NF-kB pathway by increasing reactive oxygen species [265].

BC progression can be also affected by systemic nutrients [14]. For example, meat consumption is annually rising at a global level, because meat is an important source of animal-based proteins, with superior anabolic potential when compared with plant-derived proteins [266], but it also enhances the risk of several types of cancer and other chronic diseases [267]. Lo et al. (2019) emphasized that the increased consumption of red meat was associated with invasive BC risk, which was also correlated with certain meat-cooking practices [90]. Inoue-Choi et al. (2015) showed that a higher intake of processed meat was associated with a 27% higher risk of localized postmenopausal BC and a 19% higher risk of distant BC [268]. Moreover, higher nitrite intake from processed meat was positively associated with localized cancer, whereas heme iron intake from red meat was positively correlated with BCR overall and all cancer stages [268]. Kim and Shin (2021) showed that processed meat consumption increases the risk of hypercholesterolemia, hypertriglyceridemia, and dyslipidemia, whereas red meat consumption also increases the risk of hypercholesterolemia, hyper-LDL cholesterolemia, and dyslipidemia [269], which have been linked to BC incidence [270]. Brindisi et al. (2022) demonstrated that cholesterol activates the estrogen-related receptor alpha (ERRα) pathway, promoting EMT in MCF7 and MDA-MB-231 BC cells [271]. Moreover, these authors concluded that BC cells exposed to high cholesterol levels promoted an increase in macrophage infiltration with the induction of the M2 phenotype, known as the tumor growth promoter [272], angiogenesis, and the induction of the cancer-associated fibroblast phenotype [271]. Recently, Magalhães et al. (2024) demonstrated that a high-cholesterol diet promotes phenotypic changes in BC cells and their intravasation through the LDL–LDLR axis, contributing to BC progression and metastasis in vitro and in various animal models [14]. Moreover, LDL also increases the serine proteinase inhibitor, clade E member 2 (SERPINE2) expression, which is known to be overexpressed in invasive ductal carcinoma of the breast [273]. Krawczynska et al. (2024) showed that neutrophils exposed to a cholesterol metabolite become able to secrete extracellular vesicles that promote EMT and stem cell characteristics in BC cells through the activation of the WNT/β-catenin signaling pathway, influencing BC progression [274]. Additionally, the low HDL cholesterol level was related to an increased BCR [270]. However, a cohort study in Japanese women, conducted by Narii et al. (2023), showed that triglycerides were not associated with BCR, while HDL cholesterol was inversely associated with BCR only in women over 50 years old [275].

Several pieces of analyses suggest that a higher intake of fish can be associated with higher incidence rates of ER+ BC [276], while oily fish intake has been found to be negatively correlated with the incidence of total BC, mainly in the cases of ER- BC [277]. Other authors have confirmed the protective effect of omega-3 fatty acids as a result of fish consumption against BC in Asian patients [278]. However, there are serious risks involving the consumption of fish contaminated with toxins, such as methylmercury, polychlorinated biphenyls (PCBs), dioxins, pesticides, and plastic waste [279]. Thus, food of aquatic origin is an important source of human exposure to methylmercury [280] and a low level of exposure to mercury can induce cancer cell proliferation by the estrogen receptor (ER), extracellular signal-regulated kinases 1/2 (ERK1/2), c-JUN NH(2)-terminal kinase (JNK), NADPH-oxidase, and nuclear factor erythroid 2-related factor 2 (NRF2) signaling, combined with anti-apoptotic and pro-survival signaling, the accumulation of DNA modifications, and the inhibition of DNA repair machinery [281]. Microplastics also accumulate in aquatic organisms and trigger various endocrine and metabolic pathways [282]. A study performed by Park et al. (2023) showed that polypropylene microplastics enhance metastasis-related gene expression and cytokines in BC cells [283]. Fish absorb dioxins and PCBs from their environment and these toxins enter the human body through food, including the consumption of fatty fish [284]. Invasive BCR was positively associated with dioxin exposure [285].

The various transformations involving processed meat can lead to the formation of harmful and potentially carcinogenic compounds [286]. Thus, processed meat contains polycyclic aromatic hydrocarbons (PAHs), heterocyclic aromatic amines (HAAs), residues, such as nitrosamines, biogenic amines, and a wide variety of other contaminants, such as antibiotics and other veterinary medicines and growth promoters [287,288,289,290]. Low-dose PAH-enriched mixtures have been shown to upregulate aryl hydrocarbon receptor (AhR) expression and cytochrome P450 (CYP) activity in ER+ BC cells, thus increasing cell proliferation and stimulating the expression of anti-apoptotic proteins [291]. Furthermore, a cohort study concluded that PAH exposure during pregnancy could interact with tobacco smoke, thus impacting the breast tissue in mothers and daughters, potentially influencing BCR across many generations [292]. HAAs are formed during high-temperature meat cooking and are linked to BC initiation [293,294,295]. Moreover, aromatic amines are generally highly lipophilic and can accumulate in the fatty tissue of breasts [296]. Nitrosamines are potent carcinogens formed in processed meats, when nitrites and nitrates interact with amines during food processing [297]. Studies have shown a positive association between BC and the consumption of processed meats containing these carcinogens [298]. Research indicates that consuming processed meat more than once a week is linked with a higher risk of BC, especially in women who also consume alcohol [299,300,301]. Moreover, a meta-analysis of prospective studies found that processed meat intake is associated with a 6% increase in BC development [302], while another study revealed that the said consumption is linked to a 9% higher BCR [303].

Nevertheless, milk consumption is more often associated with benefits than harm, but milk intake can be associated with a higher risk of hormone-related cancers [304]. A comprehensive meta-analysis of prospective cohort studies concluded that a high amount of whole/high-fat milk consumption can be associated with higher cancer mortality [66]. The intake of milk and dairy products has been related to a higher risk of breast and prostate cancers, due to the positive association with systemic levels of insulin-like growth factor 1 (IGF-1), insulin and estrogen signaling [305,306], and the IGF signaling pathway, implicated in the regulation of breast cancer stem cells (BCSCs), EMT, local migration and invasion, angiogenesis, and chemotherapy resistance [307]. More than 400 fatty acids (FAs) have been detected in milk; however, saturated fatty acids (SFAs) are present in a greater concentration [308]. Recently, based on a systematic review and meta-analysis, Mei et al. (2024) emphasized that high total SFA levels have been correlated with increased BCR [309]. Additionally, Jiang et al. (2024) also studied the association between dietary intake of SFAs and BCR, emphasizing that this impact depends on the carbon chain lengths of SFAs, attributable to the dietary sources and biological activities of such compounds [310].

An increased level of intake of omega-3 fatty acids associated with a decrease in omega-6, resulting in a higher omega-3/omega-6 ratio, is inversely associated with BCR [311]. Linoleic acid (LA), an omega-6 acid, predominantly present in oil seeds (soy bean, sunflower, rapeseed, and cotton), as well as α-linolenic acid (ALA), an omega-3 fatty acid found in flaxseed and fresh forage, are the FA precursors of conjugated linoleic acid (CLA) synthesis [56]. Espinosa-Neira et al. (2011) previously demonstrated that LA, an essential and the major polyunsaturated fatty acid (PUFA) in most diets, induces an EMT-like process in the mammary epithelial cells, MCF10A [312]. The authors showed that LA promotes a decrease in E-cadherin expression, as well as in increase in Snail1, Snail2, Twist1, Twist2, Sip1, vimentin, and N-cadherin expression [312]. Moreover, LA induces focal adhesion kinase (FAK) and NF-κB activation, and promotes an increase in MMP2 and MMP9 secretion, cell migration, and invasion in MCF10A cells [312]. Serna-Marquez et al. (2017) demonstrated that LA induces AKT2 activation, invasion, increases NF-κB-DNA binding activity, miR34a upregulation, and miR9 downregulation in the MDA-MB-231 BC cell line [313].

Milk also contains a plethora of proteins that may develop malignant activities. Thus, lactadherin/milk fat globule-EGR factor 8 (MFG-E8) is a glycoprotein associated with the milk fat globule membrane that plays crucial roles in cell adhesion and the promotion of angiogenesis, thus overexpressed lactadherin has been associated with poor prognosis and low survival in BC and other types of malignancies [314]. MFG-E8 can be considered as a potential biomarker and therapeutic target for breast carcinoma, emphasizing the decreased expression in ER+ and HER2+ BCs, while highly expressed in TNBC cell lines and patient sera [315]. Consequently, MFG-E8 downregulation was associated with cell cycle arrest and cell apoptosis, also inhibiting the expression of MMPs and EMT-associated proteins [315]. Osteopontin (OPN), a secreted multifunctional phosphorylated protein [60] present in milk from cows, buffalos, sheep, goats, and yaks [316], has also been identified to be highly expressed in the tumor tissue and plasma of BC patients, including TNBC tissues and cells, in association with a poor clinical prognosis and reduced survival, while OPN downregulation inhibits BC skeletal metastasis in vitro [317,318]. Recently, Guo et al. (2024) showed that OPN promotes tumor growth and metastasis and GPX4-mediated anti-lipid peroxidation in TNBC by activating the PI3K/Akt/mTOR pathway, elevating cell proliferation, invasive and migratory abilities, tumor sphere formation and angiogenesis [318]. Jia et al. (2024) showed that cottage/ricotta cheese intake was causally associated with luminal A-like BC, while the risk of ER-negative BC decreased [69]. Moreover, cow’s milk is a rich source of growth factors, including transforming growth factor (TGF)-β [319], a pluripotent cytokine with a key role in EMT, invasion, migration, and apoptosis, which acts as a growth inhibitor in early BC and a growth promoter in advanced stages of the disease [320].

Beyond all these negative effects, milk and milk-based products may contain pesticides that act as endocrine disruptors, with carcinogenic potential [67]. For example, atrazine, a herbicide that can bioaccumulate over time, was detected over the permissible limit for human consumption in bovine milk samples obtained from dairy farms [321]. Atrazine promotes 4T1 TNBC cell proliferation and migration, suppresses local and systemic immune function, and upregulates the expression of matrix metalloproteinase MMP2, MMP7, and MMP9 [322]. Moreover, atrazine induced IL-4 overexpression, while IFN-γ and TNF-α were found to be decreased in TME and serum [322]. Dairy products, including raw milk, ultra-high temperature milk, pasteurized milk, pasteurized and traditional butter, and pasteurized and traditional cheese, may also contain cypermethrin, deltamethrin, and hexachlorobenzene, over the maximum limit set by the EU [323]. Cypermethrin is a synthetic pyrethroid frequently used in agriculture and households for insect control [324]. Flumethrin, another pyrethroid pesticide, induces genotoxicity in MCF7 BC cells, even at low concentrations [325]. Hexachlorobenzene (HCB) is an organochlorine compound, which is able to bioaccumulate in high-fat tissues, that binds to the aryl hydrocarbon receptor, activating the membrane and nuclear pathways involved in BC development, such as ERα signaling, and insulin-like growth factor-1, epidermal growth factor, and transforming growth factor beta 1 receptors [326]. Thus, HCB stimulates epithelial cell proliferation, migration, invasion, and angiogenesis [326]. Unfortunately, even in the case of vegan milk substitutes, such as soy milk and its derivatives, genetically modified (GM) glyphosate-tolerant soy beans (GT), which dominate the soy bean market throughout the world, introduce thousands of tons of herbicides into the food chain [327] and bioaccumulate glyphosate (GLY), as well as its major degradation product aminomethylphosphonic acid (AMPA), which may itself act as an endocrine disruptor, mimicking 17β-estradiol that promotes ERα activity in BC cells [328]. The most common crops associated with the use of GLY include soy bean (*Glycine max*), corn (*Zea mays*), canola (*Brassica napus*), sugar beet (*Beta vulgaris*), and wheat (*Triticum aestivum*) [329]. Both GLY and AMPA have been shown to act as potential endocrine disruptors, while also exhibiting cytotoxic effects on BC cell lines. A study performed on MCF-7 and MDA-MB-468 cells highlighted the effects of these compounds on well-known signaling pathways, concluding that GLY and AMPA can dysregulate hedgehog, TGF-β, NOTCH, JAK-STAT, WNT, RAS, MAPK, and PI3K-AKT pathways, also affecting DNA repair processes, the cell cycle, and apoptosis [330], or increasing BC cell proliferation rates [328].

A literature search conducted by Keum et al. (2015) concluded that consuming more than five eggs/week was significantly associated with an increased BCR compared with no egg consumption [102]. As well as dairy products, home-grown eggs could be exposed to pesticides, such as hexachlorocyclohexane, aldrin, and malathion, more than commercial eggs, due to the direct interactions between eggs and the polluted environment [331]. Hexachlorocyclohexane has been associated with an increased BCR [332].

Augustin et al. (2013) emphasized that BCR revealed strong positive associations with bread and pasta consumption in women [93]. Pogurschi et al. (2021) analyzed the presence of acrylamide, which forms in several heated products consumed almost daily, such as coffee beverages, potato chips, pasta, pizza bases, cereal flakes and breakfast cereals, pancakes, pretzels, bread and pastries [333,334]. A large cohort study suggested a positive association between dietary acrylamide and BCR, mainly in premenopausal women, while other authors showed that acrylamide consumption did not increase the BCR [335,336]. A hormonal mode of action for acrylamide has been hypothesized to decipher the tumorigenesis in mammary glands based on acrylamide-induced DNA adduction and its mutagenesis-related potential [337].

**Figure 2:**
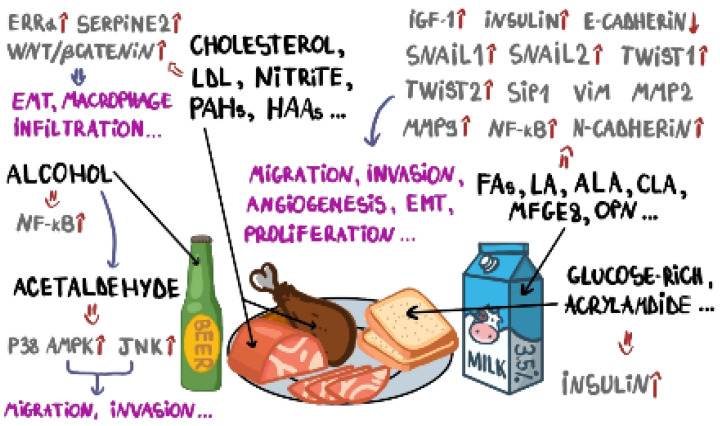
Biomarkers and molecular pathways involved in BC development, targeted by harmful compounds present in a daily diet.

Recently, a study performed by Lara-Castor et al. (2024) found that the intake of sugar-sweetened beverages among children and adolescents in 185 countries increased by 23% from 1990 to 2018 [338]. The consumption of sugar-sweetened beverages was associated with a slightly higher BCR among lean women, but no significant increases in BCR were reported overall, as well as for the consumption of artificially sweetened beverages [339]. However, there is evidence based on large-scale population-based studies that suggests that there is a positive association between a higher intake of artificial sweeteners, such as aspartame and acesulfame-K, and increased BCR [340]. Other findings, based on a systematic review and meta-analysis, confirmed that there is no association between the exposure to artificial sweeteners and the incidence of BC [341]. However, many studies sustain a direct association between sugar-sweetened beverages and weight gain, being overweight, and obesity in children and adolescents [342], whereas obesity can increase the amount of circulating proinflammatory cytokines, promote tumor angiogenesis, and stimulate cancer cell stemness, increasing BC growth, invasion, and metastasis [343]. The harmful effects, at the molecular level, of dietary compounds that exert pro-tumorigenic effects in BC are illustrated in Figure 2.

## 4. Outlook

Evidence suggests that environmental exposure to chemicals and lifestyle account for 70% to 90% of the risk factors for chronic diseases, whereas only 10% to 30% can be explained by the specific genomic landscape [2]. In this review, we highlighted both “the good” and “the bad” sides of the daily human diet or dietary patterns and behaviors that influence BC risk (BCR) and incidence. Thus, pro-inflammatory diets, based on a high intake of red, processed meat, and alcohol, have been associated with increased BCR, while diets rich in antioxidants and anti-inflammatory compounds were all correlated with a lower risk of developing BC.

Milk, meat, eggs, and bread, including their derivatives, are complex foods, rich in nutrients, considered complete and basic ingredients in almost every meal and specific dietary pattern worldwide. However, milk, bread, eggs, and meat bi-directionally impact BCR. Moreover, milk contains proteins and lipids that can induce apoptosis and BC cell cycle arrest, inhibiting invasion, metastasis, and tumor angiogenesis. However, several milk proteins, such as osteopontin, may exhibit antitumorigenic characteristics, but only in certain circumstances. In addition, osteopontin can also promote tumor growth and metastasis in TNBC, by activating the PI3K/AKT/mTOR pathway, elevating BC cell proliferation, invasion, and migratory abilities, tumor sphere formation, and angiogenesis. Lactoferrin, a natural proapoptotic iron-binding multifunctional glycoprotein from bovine milk, can exert strong anticancer activities, inducing apoptosis in highly metastatic BC cell lines. Bovine milk-derived extracellular vesicles (EVs) are able to sensitize TNBC cells to doxorubicin, but many proteins from EVs provide context-based and opposing roles, acting both as BC metastasis promoters and suppressors. The intake of milk and dairy products has been related to a higher risk of breast and prostate cancers, due to their positive association with systemic levels of insulin-like growth factor 1 (IGF-1), insulin and estrogen signaling, which are implicated in the regulation of breast cancer stem cells (BCSCs), EMT, local migration and invasion, angiogenesis, and chemotherapy resistance. Linoleic acid (LA), an essential and the major polyunsaturated fatty acid (PUFA) in most diets, induces an EMT-like process in mammary epithelial cells. Lactadherin, a glycoprotein associated with the milk fat globule membrane, plays crucial roles in cell adhesion and the promotion of angiogenesis, so overexpressed lactadherin has been associated with a poor prognosis and a low survival rate in BC. Cottage/ricotta cheese intake is causally associated with luminal A-like BC and its consumption decreases the risk of ER-negative BC, while several additives derived for saffron and used to color cheese may exert antiproliferative effects on TNBC cells. Atrazine, a herbicide that can bioaccumulate over time, detected over the permissible limit for human consumption in bovine milk, promotes TNBC cells proliferation and migration, suppresses local and systemic immune function, and upregulates the expression of matrix metalloproteinases MMP2/7/9. Hexachlorobenzene (HCB), an organochlorine compound, is able to bioaccumulate in high-fat tissues, impacting ERα signaling, insulin-like growth factor-1, the epidermal growth factor, and transforming growth factor beta 1 receptors.

Meat consumption is annually rising at the global level, but red and processed meat are associated with invasive BC risk, which is also correlated with certain meat-cooking practices. High-cholesterol-based dietary patterns promote the intravasation of BC cells, as well as the progression of BC, due to the activation of ERα signaling pathway and the development an inflammatory TME. Food of aquatic origin is an important source of human exposure to methylmercury and other endocrine disruptors and even a low level of exposure can induce BC cell proliferation through the activation of ER, ERK1/2, JNK, NADPH-oxidase, and NRF2 signaling, combined with anti-apoptotic and pro-survival signaling, the accumulation of DNA modifications, and the inhibition of DNA repair machinery. Microplastics accumulated in aquatic organisms also trigger various endocrine and metabolic pathways. Low-dose polycyclic aromatic hydrocarbon (PAH)-enriched mixtures have been shown to increase BC cell proliferation and stimulate the expression of anti-apoptotic proteins. Moreover, PAH exposure during pregnancy impacts the breast tissue in mothers and daughters, potentially influencing BCR across many generations. Heterocyclic aromatic amines (HAAs) are formed during high-temperature meat cooking, are generally highly lipophilic, accumulate in the fatty tissue of the breasts, and were linked to BC initiation. Ethanol intake, alone or combined with red and processed meat, even at low concentrations, as well as its major metabolite, acetaldehyde, causes TNBC cell proliferation, migration and invasion, as well as high levels of glucose, which increases BC cell proliferation and viability, while decreasing apoptosis and stimulating tumor angiogenesis and ROS production.

Grains are one of the most important foods consumed worldwide, which contain bioactive compounds, but the contamination of cereals and cereals-based products with mycotoxins, such as aflatoxins, has been associated with mutagenesis and carcinogenesis. Common crops are associated with the use of glyphosate (GLY) and its major degradation product, aminomethylphosphonic acid (AMPA). Even in case of vegan milk or meat substitutes, such as soy-based derivatives, genetically modified glyphosate-tolerant soy beans (GT), which dominate the soy bean market throughout the world, introduce thousands of tons of herbicides into the food chain. GLY and AMPA can dysregulate hedgehog, TGF-β, NOTCH, JAK-STAT, WNT, RAS, MAPK, and PI3K-AKT pathways, also affecting DNA repair processes, the cell cycle, and apoptosis of BC cells. Acrylamide, which forms in several heated products consumed almost daily, such as coffee beverages, potato chips, pasta, pizza bases, cereal flakes and breakfast cereals, pancakes, pretzels, bread and pastries, has a hormonal mode of action, enhancing tumorigenesis in mammary glands based on acrylamide-induced DNA adduction and its mutagenesis-related potential. Ovotransferrin, an iron-binding glycoprotein present in both avian plasma and egg white, has anticancer activities, with negative effects on the proliferation of BC cells by inducing apoptosis. However, home-grown eggs could be exposed to pesticides, such as hexachlorocyclohexane, aldrin, and malathion, more than commercial eggs, due to the direct interactions between hens or eggs and the polluted environment. Many studies claim that there is a direct association between sugar-sweetened beverages and weight gain, being overweight, and obesity in children and adolescents, whereas obesity can increase the amount of circulating proinflammatory cytokines, promote tumor angiogenesis, and stimulate cancer cell stemness, increasing BC growth, invasion, and metastasis.

However, regularly eating new, diversified, colorful, clean, nutrient-rich, energy-boosting, and raw food, enables increased apoptosis and autophagy, antioxidation, cell cycle arrest, anti-inflammation, and immune response against BC cells, the reduction or inhibition of genomic instability, BC cell stemness, growth, proliferation, invasion, migration, and metastasis. Uncontrolled growth is a hallmark of cancer and is an important event during BC development and progression. Dietary compounds can induce cell cycle arrest through the upregulation of p21, and the downregulation of CDK2/4/6, survivin/BIRC5, PCNA, cyclin D1, MARK4, and RASSF1A. For example, survivin, also known as the BIRC5 protein, is overexpressed in TNBC [344], associated with a high proliferative capacity of tumor cells, so targeting survivin/BIRC5 may be an option for patients with TNBC. A diet containing tomatoes, *Citrus* fruits, medicinal plants, and spices, rich in naringin and Rosmarinic acid, could help the induction of BC cell cycle arrest. The development of cyclin-dependent kinase (CDK1/2/4/6) inhibitors with reduced toxicity is important for improving BC patient survival outcomes. Thus, hesperetin, apigenin, daidzein, ellagic, and caffeic acids, all present in herbal teas, *Citrus* fruits, nuts, berries, spices, vegetables, green and roasted coffee, oils, honey, and propolis extracts, downregulate CDK 1/2/4/6 that, as well as cyclins, play an important role in BC cell cycle progression [345]. The cyclin-dependent kinase inhibitor 1A (p21/CIP1) also has an important role in the cell cycle by inhibiting the activity of CDKs [346]. Allicin found in garlic, hesperidin from Citrus fruits, naringin, apigenin, tangerine, daidzein, caffeic acid, malvidin, an abundant anthocyanin found in red wine and colored fruits, such as all types of berries, and kaempferol, have the ability to induce cell cycle arrest. Microtubule-affinity regulating kinase 4 (MARK4) controls the early step in cell division and migration through the Hippo signaling pathway and is considered to be a potential drug target. Rosmarinic acid shows a significant binding affinity to MARK4, inhibiting its activity [347].

Inducing apoptosis is a key strategy to control excessive BC cell proliferation and natural products possess this property, stimulating proapoptotic mechanisms, including mitochondrial functions, PI3K/AKT, ROS, and MAPK-mediated pathways [348]. Thus, bioactive compounds increase ROS, caspases 3/7/8/9, the Bax/Bcl-2 ratio, BAK, NOXA, p53, and downregulate STAT3, pAKT, mTOR, JAK2, and survivin/BIRC5. Thus, all the bioactive compounds listed in Table 1 are able to induce apoptosis. Consequently, a plant-based diet and “eating the rainbow” is important for the induction of cellular death in BC. As the hallmark of BC neoplastic behavior, metastasis is a multistep process that includes cell migration and the colonization of distant niches into target organs. The epithelial–mesenchymal transition (EMT) confers BC cells with enhanced stem cells, and invasive and metastatic proprieties, so EMT blocking is crucial to avoid the spread of BC cells [349]. Thus, everyday plant-based meals contain the phytonutrients able to downregulate mesenchymal markers and EMT-associated pathways β-catenin, vimentin, N-cadherin, MMP 2/9, PI3K/AKT, mTOR, ERK1/2, Snail1, Slug, ZEB1, overexpressing epithelial biomarkers, such as claudin, E-cadherin, and PTEN. Dietary compounds also target antioxidation through activation of the NRF/KEAP1 signaling pathway with anti-BC effects, and anti-inflammation, through the downregulation of pro-inflammatory interleukins 1/6/8, NF-kB, TNF-α, COX2, and iNOS. Autophagy can be stimulated by the upregulation of p-AMPK and the downregulation of mTOR.

Eating more frequently, reducing evening energy intake, consuming early breakfast and dinner also reduce systemic inflammation and, consequently, BCR. It is important to carry out complex studies to understand, mainly at the molecular level, the bioavailability, absorption in the small intestine, distribution, metabolism, bioaccumulation, degradation, interaction with colonic microflora, elimination, and toxicity of harmful, as well as beneficial dietary compounds, on their own or in chemical mixtures, in order to translate the results from experiments involving BC cell lines, systemic reviews, meta-analysis, and cohort studies, into the most appropriate and personalized dietary patterns for each BC patient, approaching the disease in a holistic manner. Thus, we suggest that a balanced dietary structure combined with personalized nutritional behaviors can really improve treatment efficacy and BC patient outcomes.

## 5. Conclusions

Here, we highlighted both “the good” and “the bad” sides of daily human diet or dietary patterns and behaviors that influence BC risk and incidence, and provided an outlook on the major factors that can influence the onset and/or progression of BC. Environmental exposure to chemicals and lifestyle account for 70% to 90% of the risk factors for chronic diseases, whereas only 10% to 30% can be explained by the specific genomic landscape. So, regularly eating new food allows for increased apoptosis and autophagy, antioxidation, cell cycle arrest, anti-inflammation, and immune response against BC cells, the reduction or inhibition of genomic instability, BC cell stemness, growth, proliferation, invasion, migration, and metastasis. In addition, eating more frequently, reducing evening energy intake, consuming early breakfast and dinner also reduce systemic inflammation and, consequently, BC risk. Thus, a balanced and personalized dietary structure combined with appropriate nutritional behaviors can improve treatment efficacy and BC patient outcomes.

## Figures and Tables

**Table 1:** Potential anticarcinogenic roles of phytochemicals in regard to breast cancer.

Bioactive Dietary Compounds	Food Sources	In Vitro and In Vivo Models	Effects on Molecular Biomarkers/Signaling Pathways	Effects on Biopathological Processes	Role in BC
Sulforaphane	Organo-sulfur compound obtained from broccoli/broccoli sprouts, kale, cabbage, cauliflower, garden cress, mustard [112,113]	MDA-MB-231 and MDA-MB-157 [116]	Targets MAPK/ERK [116]; downregulates NF-κB, AKT, and KEAP1; affects histone deacetylases involved in chromatin remodeling, and NRF2 antioxidant signaling [113]	Inhibits cell proliferation; causes apoptosis and cell cycle arrest; has antioxidant activities [113]; suppresses TGF-β1-induced migration, invasion, and metastasis of TNBC cells [116]	Chemoprotective [116], putative potential for BC treatment [113]
Allicin	Organo-sulfur compound from garlic (*Allium sativum*) [119]	MCF7 and HCC-70 [155], MCF7 and MDA-MB-231 [120]	Downregulates caspase 3/8/9 and Bcl-XL; upregulates NOXA, p21, and BAK expression [155]; induces p53 activation [120]	Decreases BC cell proliferation and viability and increases apoptosis, induces cell cycle arrest [120,155], improves DOX sensitivity [118]	Antitumor [155]
Quercetin	Flavonoid from fruits, vegetables (cruciferous vegetables, grapes, apples, tomatoes, blueberries), and herbal products (*Hypericum perforatum*, *Sambucus nigra*) [156]	4T1 and xenograft mouse model [125]; MCF-7 and MDA-MB-231 [124]	Suppresses IL-6/JAK2/STAT3 pathway [125], modulates the expression of caspase-3/8/9 [124]	Suppresses TNBC progression (proliferation, migration, and invasion) [125], induces apoptosis [124]	Potential anti-BC agent [124]; potential adjuvant for immune therapy in TNBC [125]
Luteolin/luteolol/ digitoflavone	Flavonoid from carrots, broccoli, celery, perilla mint leaves and seeds, apple skin, cabbages, parsley, onion leaves, thyme [157,158,159]	MDA-MB-231, BT549, and mouse model [157]; MDA-MB-231, MDA-MB-486, 4T1, BT549 [158]; MDA-MB-453 and MCF7 [160]	Reverses EMT through the suppression of β-catenin and VIM; stimulates E-cadherin and claudin; downregulates N-cadherin, Snail, and Slug; reorganizes F-actin [157]; inactivates AKT/mTOR and downregulates MMP9 through H3K27Ac and H3K56Ac [158]; upregulates miR-203, inhibits Ras/Raf/MEK/ERK, downregulates Bcl-2, upregulates Bax, impedes TGFβ1-induced EMT, decreases VIM, ZEB1, and N-cadherin, and increases E-cadherin [160]	Inhibits migration and invasion of TNBC cells [157], inhibits proliferation and metastasis, and promotes apoptosis of AR+ TNBC cells [158]	Chemopreventive and potential therapeutic agent for TNBC [157], including AR+ TNBC [158]
Hesperetin	Flavanone glycoside from *Citrus* fruits (oranges and lemons) [161]	MDA-MB-231 [161]; MCF7, including mammospheres [162,163,164]	Inhibits the Fyn/paxillin/RhoA signalling pathway [161]; activates the ASK1/JNK pathway, initiates the accumulation of ROS [162]; modulates the expression of p53, PPARG, and Notch1 [163]; downregulates CDK2/4 and cyclins, and upregulates p21^Cip1^ and p27^Kip1^, stimulates the binding of CDK4 to p21^Cip1^ [164]	Inhibits the migration and invasion induced by TGF-β1 [161]; exerts cytotoxic and proapoptotic effects [162]; inhibits BCSCs, exerts cytotoxicity on mammospheres, inhibits mammospheres, colony formation and migration, modulates cell cycle and induces apoptosis [163]; suppresses proliferation and stops the cell cycle in G1 [164]	Potential anti-BC agent, especially for TNBC [161]
Hesperidin	Flavanone from citrus fruits [165]	MCF7 cells [165], MDA-MB-231 [166], mammospheres [167], MDA-MB-231 [168], Wistar rats [169]	Suppresses AKT and NF-kB signalling, inhibits PD-L1 [166], increases p53 [167], binds to MCL-1 receptor [168], attenuates Ki67 [169]	Suppresses cell proliferation [165], inhibits cell migration and growth [166], suppresses mammospheres and colony formation, induces apoptosis [167], exerts cytotoxic effects [168]	Anti-BC activity [165,167], protective against DMBA-induced BC [169]
Naringenin	Flavanone glycoside from grapefruits, apples, onions, tea [170,171]	MDA-MB-231, Wistar rats induced with BC by DMBA [170], C57BL/6J mice induced with BC through a transplant of E0771 cells [172], Balb/c mice induced with BC through a transplant of transduced 4T1-Luc2 cells [173], MCF-7 [174]	Modulates mitochondrial-mediated pathway, upregulates caspase-3/7 [170], increases AMPK, decreases cyclin D1 [172], inhibits PKC, inhibits secretion of TGF-β1, causing its intracellular accumulation [173], inhibits PI3K and MAPK [174]	Inhibits cell proliferation and cell cycle, induces apoptosis, reduces the incidence of BC tumors [170], decreases cell viability in vivo, suppresses cell cycle progression [172], inhibits lung metastasis, increasing the survival rates of the mice [172], suppresses proliferation, impairs glucose uptake [174]	Antineoplastic agent [170], putative therapeutic option for TGF-β1 modulation [173], antiproliferative agent [174]
Naringin	Flavanone glycoside from tomatoes, grapefruits, and other *Citrus* fruits [175]	MDA-MB-231, MDA-MB-468, BT-549 [175]	Increases p21, decreases survivin/BIRC5, suppresses β-catenin pathway [176]	Inhibits cell proliferation, stimulates apoptosis [176]	Potential treatment agent for BC [176]
Apigenin	Flavone from parsley, onions, chamomile, oranges, wheat sprouts [177], celery, green peppers [178], thyme [179]	MDA-MB-453 and BT-474 [177], SK-BR-3 [177,178], MCF-7 [177,180], HBL-100 [177], MCF7-T, MCF7-F [179], MDA-MB-231, A549, SK-Hep1, nude mice [181]	Depletes HER2/neu and disrupts HER2/neu-GRP94 complex [177], modulates CDK1, p21^Cip1^, and p53 [178], induces degradation of ERα and AIB1 [179], blocks PI3K/AKT pathway [177,181] and β4 integrin function, inhibits pAKT, inhibits cell motility, migration, and invasion [181], inhibits AKT/FOXM pathway, suppresses FOXM1, and modulates ER signalling [180]	Suppresses BC cell growth, induces apoptosis [177], inhibits proliferation [180], ref. [177], activates p53-induced apoptosis [177], inhibits growth of ERα+ BC cells [179], inhibits metastasis [181]	Potential anticancer treatment [177]
Tangeretin	Flavone from lemons, oranges [182], other *Citrus* fruits [183]	MCF7, MDA-MB-468, MDA-MB-231, nude mice injected with MDA-MB-231 cells [182,184], Sprague-Dawley rats induced with DMBA [185], Wistar rats induced with DMBA [183]	Inhibits STAT3 and SOX2 pathways, decreases STAT3-DNA binding, reduces STAT3 in BCSCs [182], induces CYP1A1/CYP1B1 activity [184], decreases ROS and pro-inflammatory factors, protects against LPO [185], upregulates p53/p21, suppresses MMP2/9 and VEGF, reduces PCNA and COX2 [183]	Inhibits proliferation [182,183,184] and metastasis [183], inhibits BCSC formation, induces apoptosis, inhibits mammospheres and colony formation [182], decreases tumorigenicity and OS levels, boosts antioxidant levels [185]	Anti-BC effects
Daidzein	Isoflavone from fruits, nuts, soy beans and soy-based products [85]	MCF-7 [85,86,186], MDA-MB-453 [85], T47D [186], MCF-10DCIS [87]	Induces cell cycle arrest, inhibits cyclin D, CDK2/4, increases p21^Cip1^ and p57^Kip2^ expression, increases caspase-9 activity [85]; generates ROS, disrupts mitochondrial function [86]; inhibits TNF-α and suppresses hedgehog/Gli1 signalling [87]; upregulates Bax and downregulates Bcl-2, induces apoptosis and lowers ERα/β ratio and ROS outbursts [88]	Inhibits cell proliferation, induces apoptosis [85,86]; inhibits migration and invasion [87]	Anti-BC potential [88]
Genistein	Phytoestrogenic soy (*Glycine max*)-derived compound [81] from soy nuts, soy powder, soy milk, tofu, miso, natto [187], lupin, fava beans, kudzu, and psoralea [80]; exerts tyrosine kinase-modulating activities [84]	MCF-7 [79,81,82]; MCF7 and MDA-MB-435 transfected with human HER2 [84]; PDX mouse models for TNBC [80]	Suppresses IGF-1R/p-AKT and decreases Bcl-2/Bax [81]; downregulates NF-κB/Bcl-xL/TAp63, influences key epigenetic associated genes, genomic DNA, and histone methylation [80]; upregulates PI3K and MAPK signalling, downregulates p27 ^Kip1^ levels in ER+/HER2+ BC cells [84]	High concentrations kill MCF7 BC cells [79] or delay TNBC tumor growth [80]; inhibits proliferation/differentiation, induces apoptosis [81,82]; inhibits angiogenesis [83]; induces tamoxifen resistance and growth in ER+/HER2+ BC cells and inhibits growth of ER-/HER2+ BC cells [84]	Exhibits anticancer effects on various cancers [188]; chemoprevention in terms of BC carcinogenesis is concentration-, exposure time-, and BC subtype-dependent
Genistin	A glucoside form of genistein, readily absorbed in the intestine, found in soy beans and soy-derived foods, some legumes, and vegetables [187,189]	MCF-7, MDA-MB-231 [189]	Docks to ERα, ERβ, lowers CA 15-3 levels [190]; induces negative regulation of ERα signalling pathway, suppresses expression of oncogenic biomarkers [189]	Stimulates cell cycle arrest and apoptosis, reduces BC cell growth, proliferation, and angiogenesis [189]	Chemoprevention and therapy in terms of ER+ BCs [189]; useful for potential new drug discovery for BC management and treatment [190]
Lycopene	Major carotenoid found in tomatoes, red fruits, red carrots, watermelons, grapefruits, papayas [127]	MCF7, SK-BR-3, MDA-MB-468 [129]	Inhibits pAKT and mTOR signalling pathways, upregulates Bax [129]	Inhibits cell proliferation and cell cycle progression, initiates apoptosis [128]	Chemopreventive for TNBC [129]
Gallic acid	Hydroxybenzoic acid in fruits, vegetables, medicinal plants, such as grapes, gallnuts, pomegranates, hawthorn, tea leaves, capers [191,192,193], honey [194]	MCF7 [191], HCC1806 [195], MDA-MB-231 [196]	Suppresses PI3K/AKT/EGFR, nuclear accumulation of β-catenin [191,195], activates mitochondrial apoptosis pathways [195]	Inhibits survival of acidity-adapted BC cells and reduces metastatic characteristics induced by acidity [191], suppresses proliferation, promotes apoptosis [195] and ferroptosis [196]	Promising therapeutic agent for metastatic BC [191], antioxidant [193], suppresses TNBC progression [195]
Vanillic acid	Hydroxybenzoic acid in medicinal plants (e.g., *Angelica sinensis*), olives, cereals, whole grains, fruits, green tea, juices, berries, wines [197]	MCF7 [198]	Affects ROS pathway [198]	Generates ROS, promotes apoptosis [198]	Antiproliferative effects [198]
Protocatechuic acid	Hydroxybenzoic acid in olives (*Olea europaea*)/olive oil, hibiscus, white grape (*Vitis vinifera*) wine [199], purple rice bran extract [200], edible mushrooms (*Hydnum repandum*) [201], potatoes, onions, wheat [202]	MCF7 [199]	Reduces IL-6, IL-8, and suppresses VEGF [199]	Induces apoptosis and limits invasion and metastasis [199]	Potent anticancer agent [199], antioxidant [202]
Syringic acid	Hydroxybenzoic acid from olive oil, dates, grapes [203], foxtail millet bran (*Setaria italica*) [204]	MCF7, MDA-MB-231 [204]	Downregulates GRP78/SERBP-1/SCD1 signalling axis [204]	Antiproliferative activities [203,204]	Anti-BC agent [204], antioxidant [203]
Ellagic acid	Hydroxybenzoic acid from fruits, seeds, nuts, pomegranates, raspberries, strawberries, black raspberries, almonds, and walnuts [205]	MCF7 [205]	Regulates TGF-β/SMAD3 signalling axis, inhibits CDK6, binds to ACTN4 and induces its degradation via ubiquitin–proteasome pathway, reduces VEGFR-2 [205]	Suppresses BC cell growth, migration, invasion, metastasis, stimulates apoptosis, inhibits angiogenesis [205]	Anti-BC activities [205]
Caffeic acid	Hydroxycinnamic acid from fruits, green and roasted coffee, vegetables, tea, oils, spices [206,207], honey, and propolis extracts [194,208]	MCF7 [206]	Stimulates p53 and p21 genes, inhibits CDK2 [206], inhibits DNA methylation [208]	Induces apoptosis, cytotoxic effects, morphological changes in BC cells [206]	Putative antitumor agent [206]
Cinnamic acid	Hydroxycinnamic acid from cinnamon, grapes, tea, cocoa, spinach, celery [209]	MDA-MB-231, HEK293 [209]	Increases TNF-α-TNFR1 apoptotic pathway and caspases 8/3 [209]	Increases apoptosis and DNA damage [209]	Anti-BC agent [209]
p-Coumaric acid	Hydroxycinnamic acid from whole cereal grains, fruit, vegetables, Brazilian green propolis extracts [210]	MCF7 [210], BT20, BT549, MDA-MB-231, MDA-MB-436 TNBC [208]	Inhibits iNOS, COX-2, IL-1β, TNF-α, suppresses p-IκB, ERK1/2, blocks NF-κB and MAPKs pathways [211]	Reduces cell viability/cytotoxic effects, reverts the epigenetic silencing of the tumor suppressor *RASSF1A* [208], supports anti-inflammatory and immunomodulatory mechanisms [211]	Putative antiproliferative/anticancer agent [210,212]
Ferulic acid	Hydroxycinnamic acid from plants: ferulic (*Ferula foetida*), angelica, jujube kernel, rice bran, wheat bran [213], nuts, seeds [214]	MDA-MB-231 [215], MCF7 [216]	Regulates EMT [215]	Decreases viability and proliferation, increases apoptosis via activation of caspase-8 and -9, suppresses migration and metastasis [215,216]	Antitumor agent [215], antioxidant agent that protects DNA from OS [214,217]
Sinapic acid	Hydroxycinnamic acid from citrus fruits (oranges, grapefruits, lemons), berries; herbs (canola, mustard seed, rapeseed); cereals, wheat, rice, spices, oil seeds, vegetables, vinegar, *Salvia officinalis*, *Myristica fragrans* [218]	MCF7, T47D, MDA-MB-468, SK-BR-3 [219]	Downregulation of *VKORC1* and *KIF18B* [219]	Induces apoptosis [219]	Cytotoxic agent in regard to luminal A BC cell lines [219]
Rosmarinic acid	Hydroxycinnamic acid from medicinal plants, herbs, spices (*Boraginaceae*, *Lamiaceae*, *Labiatae*) [220]	MDA-MB-231, MDA-MB-468 TNBC [220]	Upregulates TNF, GADD45A, BNIP3, HRK, TNFRSF25, inhibits BIRC5/survivin, MARK4, hedgehog pathway and hippo signalling, decreases proliferation and migration via Bcl-2/BAX signalling pathway, inhibits NF-κB signalling [220,221]	Antiproliferation and migration/cell cycle arrest, apoptosis [220]	Anti-BC agent, antioxidant [220]
Chlorogenic acid	Hydroxycinnamic acid from fruits (apples, plums), vegetables (potatoes, eggplants), olive oil, spices, wine, coffee beans [222,223,224], honey [194]	Subcutaneous tumor mouse model of 4T1 cells [222]	Inhibits NF-κB/EMT signalling pathways [222]	Induces apoptosis, inhibits pulmonary metastasis, and improves anti-BC immunity [222]	Potential candidate for therapy of BC [222]
Avenanthramides (AVN-A, B, C)	Phenolic alkaloids found in oats (*Avena sativa*, *Poaceae*) [225]	MDA-MB-231 [226]	Activates caspase 3/7 [226]	Activates apoptosis and senescence, blocks cell proliferation, inhibits EMT and metastasis [225]	Anticancer effects [225]
Cyanidins/cyanidin 3-O-glucoside	Water-soluble anthocyanins found in leaves, petals, flowers, red fruits, blackberries, cranberries, grapes, cherries, apples, raspberries, peaches, plums, beans, red cabbage, red onions, purple sweet potatoes, carrots, avocadoes, olives [227,228]	BT474, MDA-MB-231, MCF7 [228,229]	Increases the expression of p53, Bax, caspase 3, *CYP1*, *CYP2*, and decreases *Bcl2* [228], blocks ERBB2/cSRC/FAK pathway [229]	Proapoptotic and cytotoxic effects [228], inhibits invasion and metastasis [230], anti-mutagenic and anticarcinogenic effects [231]	Anticancer agent [228]
Delphinidin	Polyphenolic natural pigment occurring in berries, eggplant, wine [232]	MDA-MB-231, BT474 [233]	Induces protective autophagy via suppression of mTOR and activation of AMPK pathway in HER2+ BC cells [233]	Inhibits proliferation [108], promotes apoptosis and autophagy [233], exerts anti-mutagenic and anticarcinogenic effects [231]	Anticancer effects [233], antioxidant [232]
Malvidin/ malvidin-3-O-glucoside	Abundant anthocyanin in red wine, red grapes (*Vitis vinifera*), the skin of colored fruits, blueberries (*Vaccinium corymbosum*), blackberries (*Rubus* sp.) bilberries (*Vaccinium myrtillus*), red raspberries (*Rubus idaeus*), black raspberries (*Rubus occidentalis*), cranberries (*Vaccinium macrocarpon*), strawberries (*Fragaria ananassa*) [234]	MCF7	Increases p21, caspases 3/8/9, Bax/Bcl-2, inhibits NF-κB, PI3K, TNF-α, STAT3, MMP2/9, IL-6, WNT, Notch1, and cyclin D1 [234]	Induces cell cycle arrest, antioxidation, anti-inflammation, autophagy, and apoptosis; inhibits proliferation, metastasis/cell invasion [234]	Anticarcinogenic potential [234]
Peonidin	Anthocyanidin found in purple sweet potatoes (*Ipomoea batatas*) [235], pigmented rice (red, black, dark purple) [230]	In silico [235]	Inhibits the overexpression of HER2 protein [235]	Proapoptotic, antiproliferative, anti-metastasis role [230]	Anti-BC activity [235]
Resveratrol	Non-flavonoid polyphenol from blueberries, grapes, red wine, raspberries, mulberries, apples, pomegranates, soy beans, peanuts	MDA-MB-231 [135]	Modulates PI3K/AKT, NF-κB, and Notch signalling pathways [133,134]	Induces Bax-dependent, but p53-independent, apoptosis [135]	Chemopreventive and putative therapeutic agent [236]
Curcumin	Polyphenol derived from turmeric (*Curcuma longa*)	MCF7 [137,138], MDA-MB-231, and Hs 578T [147]	Modulates NF-κB [137]; downregulates oncogenic RAF-1, suppresses telomerase, upregulates TNF-α and IL-8 genes [138]; inhibits EMT through downregulation of mTOR and PI3K/AKT signaling [147]	Inhibits cell stemness [148], proliferation, and promotes apoptosis [137]; suppresses motility and metastasis in TNBC [147]	Chemopreventive agent [137], anticancer and cytotoxic properties [138]; potential therapeutic agent [147]
Epicatechin	Flavan-3-ol from green tea, cocoa, grapes, apricots, green algae [237]	4T1 [237], [238], TNBC mice model [150], MCF-7 [239] [240], MDA-MB-468 [153], MDA-MB-231 [240]	Increases Bax/Bcl-2 ratio, increases the expression of CDH1, MTSS1, PTEN, BMRS, FAT1, and SMAD4 [237], modulates the AMPK and Akt/mTOR pathways [238]	Decreases cell growth [150,238], inhibits metastasis-associated proliferation, reduces migration [150], cytostatic effects at lower concentrations [239], inhibits proliferation [238], proapoptotic [153,240]	Antiproliferative agent, similar effects to doxorubicin in terms of tumor growth inhibition and survival rates [238], could be used as an inhibitor for BC progression (anti-metastatic, anti-migratory, anti-invasion) [150]
Catechins	Flavan-3-ols found in black grapes, strawberries, cider, red algae, green algae, red wines, kiwis, green tea, gooseberries [241,242]	4T1 [241]	Downregulates EGFR, APP, Bcl-2, DNMT, HIF1a, and PSMB5; upregulates caspase 3 and GADD45b [241]	Suppresses proliferation, stimulates apoptosis [241]	Antiproliferative agent [241,242]
Epigallocatechin gallate	Flavan-3-ol from green and black tea, apples, cherries, red algae, other fruits and vegetables [151,208,242]	BT20, BT549, MDA-MB-436, MDA-MB-231, MCF7, T47D, Hs 578T, allograft Balb/c model [151,208]	Downregulates mTOR, PI3K/AKT, p53/Bcl-2, EGFR, VEGF, STAT3, NF-κB, SCUBE2, TIMP3, DNMT, ERα; activates JNK, caspases 9/3 [151,208]	Decreases cell growth, increases apoptosis, prevents DNA damage and proliferation, inhibits invasion, reduces cell viability, has cytotoxic effects [208]	Antiproliferative and anti-invasion agent, hypomethylating agent [208]
Theaflavin	Antioxidant polyphenol found in black tea [152,243]	T47D, MDA-MB-231 [244], MCF-7, ZR-75-1 [245]	Upregulates Fas/caspase 8, downregulates pAKT/pBAD pathway [244]; increases p53, Bax, activates caspase 6/7/9, increases ROS, stimulates p53, downregulates MMP2 and MMP9, inhibits the translocation of NF-kB/p65 to the nucleus [245]	Induces apoptosis [244], probably in a p-53-dependent manner [152], reduces cell viability, inhibits cell migration, could induce p53 phosphorylation of the Ser15 residue [245]	Proapoptotic agent [244]
Theaflavin-3-gallate	Polyphenol from fruits and veggies	MCF-7, MCF-10A [246]	Downregulates HSP90, MMP9, VEGFA, and SPP1 genes [246]	Inhibits cell proliferation, no cytotoxic effects on non-malignant breast cells (MCF-10A), induces apoptosis by stopping the cell cycle in the G2/M phase, decreases migration and colony formation [246]	Potentiate other BC therapies [246]
Phlorizin	Bioactive chalcone found in *Asteraceae*, *Ericaceae*, *Saxifragaceae*, *Proteaceae*, *Rosaceae*, *Rutaceae*, *Fabaceae*, *Lamiaceae*, *Plantaginaceae*, *Pyrus communis* [247]	MDA-MB-231, T47D [248], MCF7 [249]	Inhibits ERα signalling pathway, increases apoptotic caspase 3 via p53 [249]	Stimulates apoptosis, induces cytotoxicity/genotoxicity [248,249], antioxidant, anti-inflammatory, affects the composition of gut microbiota and development [250]	Anti-BC potential [247]
Kaempferol	Flavonoid from plants, fruits, vegetables, onions, apples, berries, tea [124,251]	MCF7 [252,253,254], MDA-MB-231, xenograft models [124,255]	Suppresses cyclin D1, p21, TWIST, and p38 MAPK [252], downregulates SNAI2, PLAU, CSF1, inhibits IGF1/IGF1R-mediated EMT [255]	Induces apoptosis, inhibits growth, migration, and proliferation of BC cells [124,252,256]	Anticancer effects [124,252], potentiates sensitivity to chemotherapy drugs [253,254]

## Abbreviations

AMPK—monophosphate-activated protein kinase; APP—amyloid-beta precursor; ASK1—apoptosis signal-regulating kinase 1; BAD—Bcl-2-associated death promoter; Bax—Bcl-2-associated X protein, apoptosis regulator; Bcl-2—B-cell lymphoma 2 protein; BCSCs—breast cancer stem cells; BNIP3—Bcl-2 interacting protein 3; CDKs—cyclin-dependent kinases; COX2—cyclooxygenase 2; CSF1—colony-stimulating factor 1; DNMT1—DNA (cytosine-5)-methyltransferase 1; EGF—epidermal growth factor; EGFR—epidermal growth factor receptor; EMT—epithelial-mesenchymal transition; ERα—estrogen receptor alpha; ERK1/2—extracellular signal-regulated protein kinase 1/2; GADD45b—growth arrest and DNA damage-inducible beta; GRP78—glucose regulated protein 78; HER2—epidermal growth factor receptor 2; HIF1—hypoxia-inducible factor 1; HRK—harakiri; IGF1—insulin growth factor 1; IGF1R—insulin-like growth factor-1 receptor; IκB—IkappaB kinase; IL-6—interleukin 6; iNOS—inducible nitric oxide synthase; JAK2—Janus kinase 2; JNK—c-Jun N-terminal kinase ; KIF18B—kinesin family member 18B; KEAP1—Kelch-like ECH-associated protein 1; MAPK—mitogen-activated protein kinase; MARK4—microtubule affinity-regulating kinase 4; MCL1—myeloid cell leukemia 1; MMP-2—matrix metalloproteinase 2; MMP-9—matrix metalloproteinase 9; mTOR—mammalian target of rapamycin; MTSS1—metastasis suppressor protein 1; NF-κB—nuclear factor kappa light chain enhancer of activated B cells; NOTCH1—neutrogenic locus notch homolog protein 1; NRF2—nuclear factor erythroid 2-related factor 2; PCNA—proliferating-cell nuclear antigen; PD-L1—programmed death-ligand 1; pAKT—phosphorylated serine/threonine-specific protein kinase; pERK-phosphorylated-extracellular signal-regulated kinase; PI3K –phosphatidylinositol-4,5-bisphosphate 3-kinase; PPARG—peroxisome proliferator-activated receptor gamma; PSMB5—proteasome subunit beta type-5; p27/Kip1—cyclin-dependent kinase inhibitor 1B; PDX—patient-derived xenograft; PLAU—plasminogen activator, urokinase; PTEN—phosphatase and TENsin homolog deleted on chromosome 10; RASSF1A—Ras association domain family protein 1 isoform A; ROS—reactive oxygen species; SCD—stearoyl-CoA 9-desaturase; SCUBE2—signal peptide, CUB domain and EGF-like domain containing 2; SERBP1—SERPINE1 mRNA-binding protein 1; SLUG—zinc finger protein SNAI2; STAT3—signal transducer and activator of transcription; TGF-β1—transforming growth factor beta 1; TIMP3—TIMP metallopeptidase inhibitor 3; TME—tumor microenvironment; TNBC—triple negative breast cancer; TNF—tumor necrosis factor; TNFRSF25—tumor necrosis factor receptor superfamily 25; VEGF—vascular endothelial growth factor; VIM—vimentin; VKORC1—vitamin K epoxide reductase complex subunit 1, WNT—wingless-related interaction site.
